# Modulation of Lipid Droplet Metabolism—A Potential Target for Therapeutic Intervention in *Flaviviridae* Infections

**DOI:** 10.3389/fmicb.2017.02286

**Published:** 2017-11-28

**Authors:** Jingshu Zhang, Yun Lan, Sumana Sanyal

**Affiliations:** ^1^HKU-Pasteur Research Pole, School of Public Health, Li Ka Shing Faculty of Medicine, University of Hong Kong, Hong Kong, China; ^2^School of Biomedical Sciences, Li Ka Shing Faculty of Medicine, University of Hong Kong, Hong Kong, China

**Keywords:** lipid droplet, lipid metabolism, HCV, flavivirus, dengue

## Abstract

Lipid droplets (LDs) are endoplasmic reticulum (ER)-related dynamic organelles that store and regulate fatty acids and neutral lipids. They play a central role in cellular energy storage, lipid metabolism and cellular homeostasis. It has become evident that viruses have co-evolved in order to exploit host lipid metabolic pathways. This is especially characteristic of the *Flaviviridae* family, including hepatitis C virus (HCV) and several flaviviruses. Devoid of an appropriate lipid biosynthetic machinery of their own, these single-strand positive-sense RNA viruses can induce dramatic changes in host metabolic pathways to establish a favorable environment for viral multiplication and acquire essential components to facilitate their assembly and traffic. Here we have reviewed the current knowledge on the intracellular life cycle of those from the *Flaviviridae* family, with particular emphasis on HCV and dengue virus (DENV), and their association with the biosynthesis and metabolism of LDs, with the aim to identify potential antiviral targets for development of novel therapeutic interventions.

## Introduction

Cellular homeostasis is maintained by a constant metabolic energy flux. As one of the major energy sources, lipids are synthesized, modified and utilized through various pathways. Lipid droplets (LDs) are ubiquitous and conserved cytoplasmic compartments delineated by a phospholipid monolayer, and serve as energy reservoirs in almost all living organisms. Excess lipids are packaged, stored and distributed in LDs, an organelle which is not only important in lipid storage and metabolism, but protein quality control, pathogenesis, and immune responses (Walther and Farese, 2012).

Since viruses lack the appropriate machinery to conduct their own lipid synthesis, most have evolved mechanisms to hijack host lipid metabolic pathways (including LDs) for completing their intracellular replication cycles. Hepatitis C virus (HCV) has long been demonstrated to do so (Paul et al., 2014). Apart from the cell biology underlying infection, the interplay between viral infection and host lipid metabolic pathways is important not only to elucidate the pathogenicity of this category of viruses but also to assess how they can be targeted as a general means of combating infections.

As a consequence of development of gene editing and mass spectrometry based lipidomics and proteomics technologies, an increasing body of evidence indicates the involvement of host LDs at different steps of the intracellular life cycle of HCV and flaviviruses (Martín-Acebes et al., 2016b). Here, we have cataloged these interactions and anticipate that this knowledge will be beneficial for identification of host factors as suitable targets for antiviral interventions.

## Lipid droplet—a multifunctional organelle

### Morphology and composition of LDs

LDs are essentially the emulsion phase of insoluble oil droplets dispersed in aqueous cytoplasm. Compared to other cellular organelles with double-layered membranes, the structure of LDs is rather unique, containing a hydrophobic core and a single layer of amphipathic phospholipids. The neutral lipid core contains predominantly triacylglycerols (TAGs) and cholesterol esters (CEs) (Thiam et al., 2013). Although the composition of the phospholipid monolayer varies in different cell types, phosphatidylcholine (PC) and phosphatidylethanolamine (PE) are the two major phospholipids. The morphology and consumption of LDs are drastically altered by the composition of their phospholipid monolayer (Guo et al., 2008). The surface of the monolayer is decorated with LD-associated proteins, including lipolytic enzymes such as hormone-sensitive lipase (HSL), adipose triglyceride lipase (ATGL), and PAT-domain family (**p**erilipin, **a**dipophilin and **T**IP47) (Tauchi-Sato et al., 2002; Ohsaki et al., 2006; Wilfling et al., 2014a). Despite being present in nearly all cell types across different organisms, LDs are highly heterogeneous and dynamic with varied numbers and sizes (ranging from 100 nm to 100 mm in diameter) in otherwise identical cells. Even within the same cell, LDs expand or shrink in response to cellular signals.

### Biogenesis of LDs

In eukaryotes, LDs respond to increased cellular fatty acid levels and emerge from the accumulation of neutral lipids in the ER, which harbors enzymes necessary for neutral lipid synthesis in most cell types (Buhman et al., 2001; Pol et al., 2004). First established as an oil-in-water emulsion, the small nascent LDs undergo a series of well-organized processes and grow into larger, mature LDs. The final steps of TAG and CE synthesis are catalyzed by ER-localized diacylglycerol acyltransferases (DGATs) and acyl-CoA:cholesterol acyltranserases (ACATs), respectively. The continuous accumulation of the newly synthesized TAGs and CEs at specific sites at the ER results in separation of two phases, where globules of TAGs arise between the two leaflets of the bilayer and eventually dissociate. DGAT2, which is inserted into one leaflet of the ER membrane, is transported to LDs where it continues to catalyze synthesis of TAGs, hence promoting further growth of LDs (Kassan et al., 2013; Wilfling et al., 2013). This process is thermodynamically enabled by the unique phospholipid monolayer structure of LDs.

### The multifunctionality of LDs

Long been regarded as simple and passive lipid storage compartments, LDs are currently considered highly dynamic and complex. They play a central role in lipid metabolism and are connected to diverse cellular processes like fatty acid trafficking, cellular signaling, protein storage, autophagy, immunity, and virus replication (Singh et al., 2009; Saka and Valdivia, 2012; Rambold et al., 2015; Welte, 2015; Velázquez et al., 2016).

#### LDs as the central regulator for cellular homeostasis

As metabolically active organelles, LDs regulate the balance between host lipid synthesis and mobilization to maintain cellular homeostasis. Catalyzed by DGAT1 and DGAT2, cellular fatty acids together with diacylyglycerols (DAGs) are converted into TAGs and stored in LDs. TAGs can be further hydrolyzed to generate DAGs or phosphatidic acid (PA) for membrane phospholipid synthesis and free fatty acids (FFAs) for energy production (Pol et al., 2014).

#### LDs as transient protein storage compartments for degradation

Due to unique structural features and proximity to the ER, the surface of LDs can also serve as transient storage depots for proteins that are destined for degradation via the ER-associated degradation (ERAD) pathway (Gao and Goodman, 2015). Misfolded proteins in the ER are removed and degraded by the ubiquitin–proteasome system. Current evidence suggests that ubiquitinated apolipoprotein B100 (ApoB100) (Ohsaki et al., 2008) and 3-hydroxy-3-methylglutaryl CoA reductase (HMGCR) (Hartman et al., 2010) are likely degraded on the surface of LDs through proteasomal and autophagic pathways (Ohsaki et al., 2006). HMGCR is one of the rate-limiting enzymes for cholesterol synthesis in mammalian cells. Ubiquitination of HMGCR is mediated by ancient ubiquitous protein 1 (AUP1), a highly conserved monotopic membrane protein localized to both LDs and the ER membrane (Spandl et al., 2011). AUP1 recruits the ubiquitin-conjugating enzyme UBE2G2 to LDs and facilitates its binding with the ER ubiquitin ligases gp78 and Trc8, which subsequently initiates the ubiquitination/degradation of HMGCR resulting in inhibition of cholesterol synthesis (Jo et al., 2013). Apart from providing a molecular link between LDs and the ubiquitination machinery, monoubiquitinated AUP1 was reported to induce LD clustering, a widespread phenomenon observed in multiple cell types across all species (Lohmann et al., 2013). LDs may also provide sequestration platforms for protein storage (Cermelli et al., 2006), such as during the synthesis of eicosanoids, a class of signaling molecules that use LDs as distinct sites for eicosanoid generation (Bozza et al., 2011).

### Mobilization of lipids from LDs

Depending on the cell type, starvation and/or physiological conditions, eukaryotic cells mobilize lipids stored in LDs via two major pathways termed lipolysis and lipophagy. In mammalian adipocytes, lipolysis is activated in response to changes in cellular energy and hormone levels. This allows transient docking and activation of three major lipolytic enzymes, ATGL, HSL, and monoglyceride lipase (MGL) which co-ordinate the hydrolysis of TAGs for energy production (Karlsson et al., 1997; Zimmermann et al., 2004; Dugail and Hajduch, 2007; Lass et al., 2011). Perilipins localize to LD surfaces and under basal conditions shield TAGs from cytosolic lipases. During starvation, perilipins are degraded via the chaperone-mediated autophagy (CMA) pathway to facilitate lipolysis by HSL and ATGL (Brasaemle, 2007; Sztalryd and Kimmel, 2014; Kaushik and Cuervo, 2015). Apart from LD-associated proteins, the ADP-ribosylation factor-coat protein I (ARF1-COPI) vesicular trafficking machinery is likely to play an important role in mediating lipolysis by regulating LD composition and targeting ATGL to LDs (Soni et al., 2009; Wilfling et al., 2014b).

The role of autophagy in regulating lipid metabolism has been intensively studied in recent years (Singh et al., 2009; Singh and Cuervo, 2012). Various cell types have been used to demonstrate the process of LD mobilization via the autophagy pathway, such as hypothalamic neurons (Kaushik et al., 2011), glial cells (Martinez-Vicente et al., 2010), and enterocytes (Narabayashi et al., 2015). Autophagy is a conserved cellular process that delivers cytoplasmic contents, including dysfunctional proteins, and excess or damaged organelles to lytic compartments for degradation and recycling. The process can be induced by a number of factors such as ER stress, cellular starvation, and pathogenic infection. Available data support that three distinct types of autophagy can be triggered: macro-, micro- and chaperone-mediated autophagy, amongst which, macroautophagy is the best characterized (Yoshimori, 2004; Mizushima, 2007). Upon activation, cytoplasmic components are first enclosed by a double-layered vesicular structure termed autophagosome, which fuse with lysosomes where internal cargos are degraded (Mizushima, 2007). Multiple factors such as nutrient deprivation, virus infection, and sterol (cholesterol) depletion, can trigger degradation of LDs through the autophagic machinery (Ouimet and Marcel, 2012). LC3II, a structural component of the autophagosomes, and autophagy-related proteins Atg2, Atg5, and Atg7 are recruited to the surface of LDs to form autophagosomes. LDs are engulfed for lysosomal degradation to release stored lipids, which undergo mitochondrial β-oxidation for energy production. This process is frequently manipulated by flaviviruses to promote their replication (see Usage of LD as an energy reservoir during viral life cycle) (Singh et al., 2009; Heaton and Randall, 2010; Fujimoto and Parton, 2011; Velikkakath et al., 2012). The level and distribution of cellular cholesterol is tightly regulated; excess free cholesterol stored as cholesteryl esters in LDs are hydrolyzed during sterol starvation through autophagy (Cheng et al., 2006; Ouimet and Marcel, 2012). Sterol regulatory element-binding proteins (SREBPs) are the central transcriptional regulators of cholesterol metabolism and lipogenesis. In the presence of high cholesterol content in the cytoplasm, SREBP binds to sterol regulatory element-binding protein cleavage-activating protein (SCAP) and the ER-associated protein Insig. Upon reduction of cellular cholesterol below a threshold, Insig is degraded through the ERAD pathway, the SCAP-SREBP complex is transported to the Golgi, where SREBP undergoes intramembrane proteolysis and translocates to the nucleus. This mature form of SREBP initiates transcription of a series of down-stream genes involved in the biosynthesis of cholesterol (Brown and Goldstein, 1997; Yang et al., 2002).

## The *Flaviviridae* family

Viruses of the *Flaviviridae* family are enveloped single-strand positive-sense RNA viruses, with the nucleocapsids surrounded by two or more types of envelope glycoproteins and lipid bilayers (Lindenbach et al., 2007; Paul and Bartenschlager, 2015). It comprises several different genera including *Hepacivirus* (e.g., HCV), *Flavivirus* [e.g., Zika virus (ZIKV), dengue virus (DENV)], *Pegivirus*, and *Pestivirus*.

Persistent infection with HCV in humans can develop into serious liver diseases, including fibrosis and liver cirrhosis, which could further progress into hepatocellular carcinoma (Bartenschlager et al., 2013). Medically-relevant flaviviruses, including yellow fever virus (YFV), ZIKV, DENV, West Nile virus (WNV), and Japanese encephalitis virus (JEV), are usually arboviruses (viz., transmitted by arthropods, mainly mosquitoes and ticks) that are responsible for severe mortality in humans and animals worldwide. DENV and YFV infections are known to cause vascular leakage and hemorrhage in some infected patients (Siqueira et al., 2005; Garske et al., 2014; Thanachartwet et al., 2015). JEV and WSN infections on the other hand, tend to cause neurological diseases (Sarkari et al., 2012; Samaan et al., 2016). ZIKV infection is associated with serious birth defects—microcephaly in particular—and other neurological disorders (Petersen et al., 2016). Although there has been significant progress in therapeutic interventions for HCV and some other flaviviruses (for example YFV), there is still an urgent need for vaccines and medications against others such as DENV and ZIKV. Additionally, the ever-increasing geographical spread and number of outbreaks caused by these pathogens pose a considerable threat to public health (Gould and Solomon, 2008).

**Figure 1:**
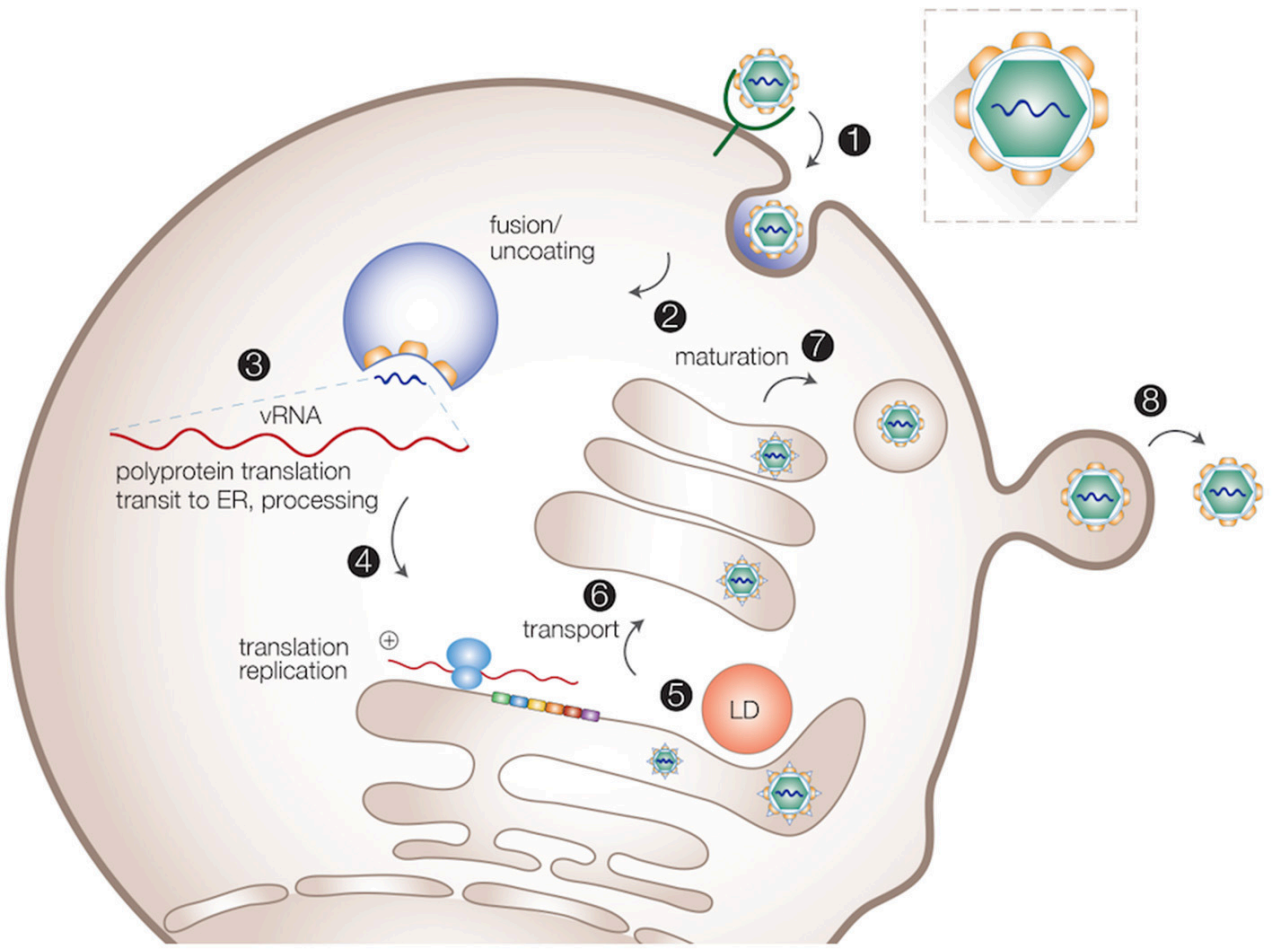
Intracellular life cycle of flaviviruses. Viral particles are internalized via receptor-mediated endocytosis (1). After the uncoating of viral particles (2), viral RNA is released into cytosol for translation and replication (3,4). Progeny virions are assembled in close proximity to the ER and LDs (5). Virions are transported through the host secretory pathway and undergo maturation (6,7). Mature virions are released from the cell surface (8).

Despite significant differences in transmission, tissue tropism and pathogenesis, viruses of the *Flaviviridae* family employ similar intracellular replication strategies. After receptor-mediated endocytosis, the acidic environment in the endosomes triggers fusion between the virion lipid envelope and cellular membranes, followed by viral uncoating. The viral RNA is subsequently released into the cytoplasm and used directly as mRNA for translation of the viral polyprotein. Host and viral proteases cleave the newly synthesized viral polyprotein to generate the structural and non-structural (NS) proteins (Lindenbach et al., 2007). Viral replicase proteins together with other host factors induce massive rearrangements of intracellular membranes to form organelle-like membrane-delineated compartments for efficient RNA replication. At the replication sites, the positive-sense RNA is used as template to generate the negative-sense RNA intermediate, while multiple positive-sense progeny RNAs are produced to be incorporated into nascent virus particles (Paul and Bartenschlager, 2015). Progeny virions are assembled in close proximity to the ER and LDs, and appear to bud into the ER-lumen, followed by transport through the host secretory pathway where they undergo further maturation, and are eventually released from the cell surface (Lindenbach et al., 2007; Paul and Bartenschlager, 2015; Figure 1).

## Influence of LD metabolism on the virus life cycle

HCV has historically been used for studying interactions between LD metabolism and the viral life cycle. Others from the same family, such as DENV, have recently started receiving more attention in this regard. The magnitude and complexity of these interactions underscore the significance of targeting LD metabolism to control viral infection. As a dynamic cellular lipid storage organelle, LDs participate in the viral life cycle by providing intracellular membrane surface area, lipids, energy, and proteins.

### Contribution of LDs in virus replication and assembly

**Figure 2:**
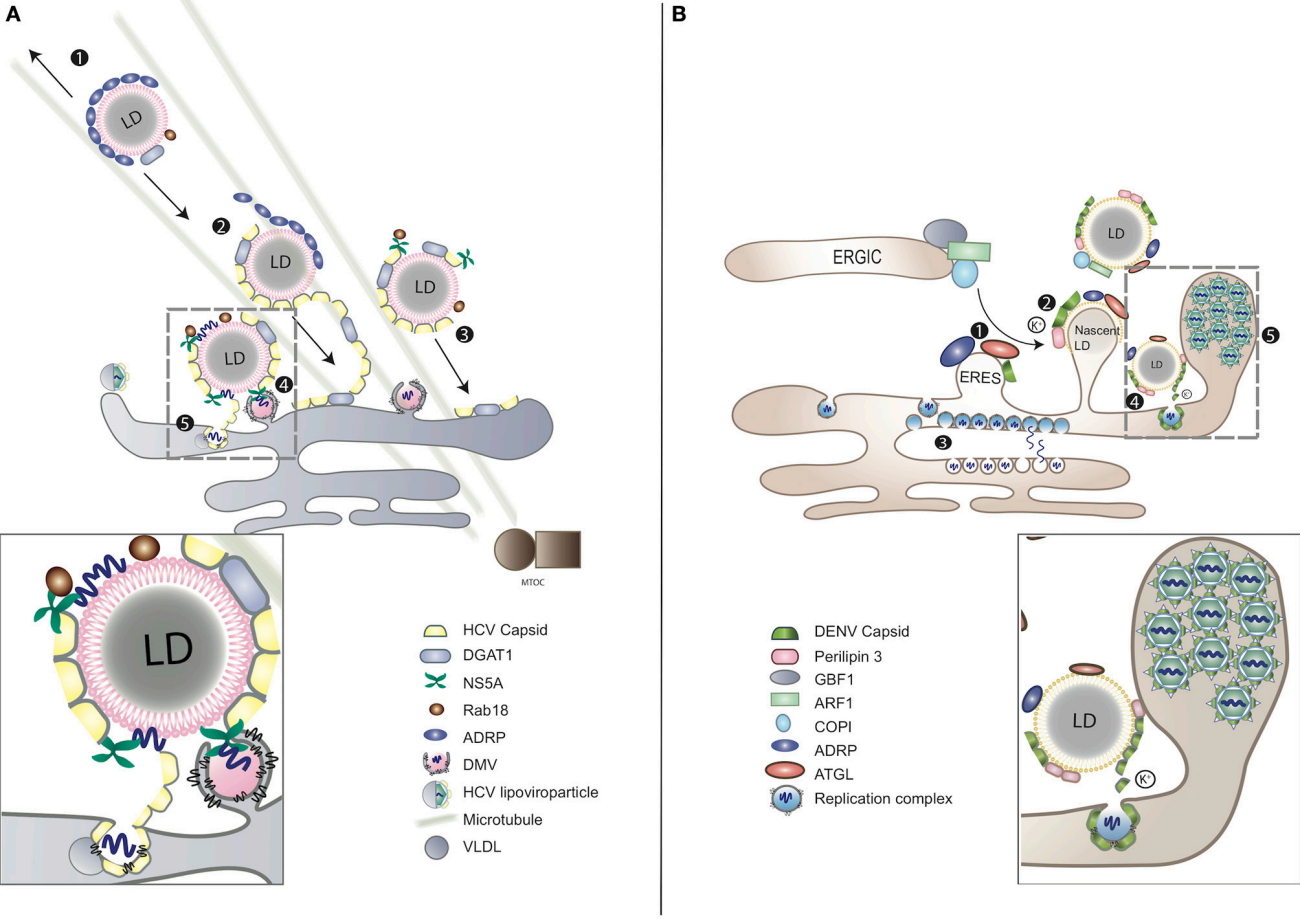
LDs as platforms for virion assembly in **(A)** HCV and **(B)** DENV infection. **(A)** (1) ADRP-coated LDs are able to interact with microtubules and move toward both plus and minus ends. (2) During HCV infection, viral capsid protein replaces ADRP from LD surface with the assistance of DGAT1. (3) As the consequence of losing ADRP, LD losses the balance of mobility, moving toward MTOC and nucleus. (4) Clustering of LDs at the peripheral of nucleus enables the contact between LDs and the replication complex of HCV. HCV RNA is delivered from ER-bound replication complexes to NS5A, obtaining access to LD surface, followed by nucleocapsid formation (gray-dashed frame and enlarged panel). (5) The nucleocapsid fuses with VLDL to form viral lipoviroparticle in ER. **(B)** (1) At the ER–Golgi intermediate compartment (ERGIC), ARF1 and its guanine nucleotide exchange factor (GEF) GBF1 together with COPI deliver ATGL and ADRP from ER export sites (ERES) to the surface of LD. DENV subverts this process for the transportation of capsid protein to LD surface. (2) The accumulation of DENV capsid protein on LDs associates with cellular perilipin 3 and intracellular K^+^ concentration. (3) Replicated DENV genomes are released through the vesicle pore and then engaged into nucleocapsids that bud through the ER membrane in close proximity. (4) Capsid protein can be released from LDs to the cytosol or other cellular compartments for subsequent viral assembly (gray-dashed frame and enlarged panel). (5) Packed virions accumulate within the lumen of the vesicle packets-containing ER network before transported to Golgi (Boulant et al., 2008; Chatel-Chaix and Bartenschlager, 2014).

Upon infection massive intracellular membrane rearrangements are induced by perturbing lipid biosynthetic pathways to spatially segregate the replication and assembly sites (Welsch et al., 2009; Romero-Brey et al., 2012). On the one hand, the two sites need to be separated to avoid competition between the capsid protein and the viral replicase complex at the level of RNA binding. On the other hand, newly synthesized positive-sense progeny RNAs need to be transported from the replication to the assembly sites, where the capsid protein is concentrated. For maximum efficiency in virus assembly the two sites require close proximity to each other (Welsch et al., 2009; Romero-Brey et al., 2012; Figure 2).

#### Association of LDs to viral replication compartments

LDs have been reported to associate with virus-induced membrane bound compartments believed to be replication sites. Despite belonging to the same family, HCV and DENV induce morphologically distinctive replication compartments. In the case of HCV infection, the double-membrane vesicles (DMVs) are derived from the ER (Romero-Brey et al., 2012; Figure 2A). DMVs are composed of active viral replicase proteins and double-stranded RNA (dsRNA), along with several host components including vesicle-associated membrane protein-associated protein A (VAP-A) and VAP-B that are crucial for viral RNA replication (Evans et al., 2004; Gao et al., 2004). The highly hydrophobic NS4B of HCV, together with NS5A, are the major viral factors that contribute to DMV formation (Lundin et al., 2006). These virus-induced compartments use cholesterol as a structural component and can be visualized in close proximity to LDs (Romero-Brey et al., 2012; Paul et al., 2013). While DMVs are considered as replication factories of HCV, their association to LDs is still unclear. The interferon-induced antiviral protein viperin, which inhibits HCV RNA replication, localizes to LDs using a similar mechanism as HCV NS5A, indicating the importance of LD-NS5A association during HCV replication (Jiang et al., 2008; Hinson and Cresswell, 2009). LDs release free cholesterol from the esterified form for membrane biogenesis as per the host cellular requirements (Maxfield and Tabas, 2005) and, therefore, may serve as reservoirs for lipids required for expanding the intracellular membrane surface to form DMVs (see Association of LDs to Viral Replication Compartments). Besides, HCV replication triggers the activation of the cellular SREBP pathway for *de novo* synthesis of membrane lipids, which, in turn, regulate biogenesis of LDs (see Manipulation of LD Reserves during Viral life Cycle) (Li et al., 2013). Another possibility is that LDs themselves provide a platform for virus assembly and, therefore, require close proximity to the replication sites for efficient recruitment of newly synthesized viral proteins and subsequent virion packaging (see LDs as a Platform for Virion Assembly) (Miyanari et al., 2007).

Unlike HCV, DENV infection induces formation of single-membrane in-folding into the ER lumen and unstructured convoluted membranes (Welsch et al., 2009; Figure 2B). These DENV-induced vesicle-like structures contain viral replicase and dsRNA. Pore-like openings on these structures enable release of newly synthesized viral RNA, facilitating replication and efficient encapsidation (Welsch et al., 2009). Other flaviviruses, such as WNV and tick-borne encephalitis virus (TBEV) share similar features of intracellular membrane rearrangements (Gillespie et al., 2010; Miorin et al., 2013). DENV replication activates the autophagy pathway to mobilize FFAs from LDs and co-opts FA synthase (FASN). FFAs released from LDs are consumed by oxidation in mitochondria to generate ATP, which is required for viral RNA replication (see Usage of LD as an Energy Reservoir during Viral life Cycle) (Heaton and Randall, 2010). Moreover, DENV NS3 recruits FASN to virus replication sites during membrane remodeling in a Rab18-dependent fashion, engaging both LDs and the viral replication complexes in the process (Heaton et al., 2010; Tang et al., 2014). Regardless of the distinct membrane compartmentalization strategies of HCV and DENV both require close juxtaposition of LDs for energy supply and subsequent virion assembly, as reviewed below.

#### LDs as a platform for virion assembly

In the case of HCV infection, after being generated at the ER, the capsid protein localizes to LDs via its domain 2 in a time-dependent manner. They accumulate on discrete regions of LDs before fully covering the surface of LDs (Boulant et al., 2007; Shavinskaya et al., 2007). Host DGAT1 that synthesizes triglycerides stored within LDs, binds to the HCV capsid protein, which in turn acquires access to DGAT1-generated LDs. Viral RNA replication complexes are subsequently recruited to appropriate sites of virus assembly. LD-localized capsid protein provides stability to these structures via interfering with TAG turnover and inducing aggregation of LDs (Boulant et al., 2008; Herker et al., 2010; Harris et al., 2011). Additionally, by replacing LD-localized ADRP, the capsid protein induces imbalance between the minus-end-directed and the plus-end-directed motors, causing movement of LDs on microtubules toward the nucleus so as to enhance interactions between sites of HCV RNA replication and virion assembly (Boulant et al., 2008). The capsid protein recruits viral NS5A, while the N-terminal of NS5A engages viral replication complexes to LD-associated membranes (Boulant et al., 2007; Appel et al., 2008). HCV NS5A also associates with Rab18, a member of the Rab GTPase family that plays an essential role in membrane traffic (Salloum et al., 2013). Rab18 localizes directly to the monolayer surface of LDs in response to lipolytic stimulation (Martin et al., 2005), and facilitates association of NS5A and other replicase components with LDs (Salloum et al., 2013; Figure 2A). HCV infection increases the expression of apolipoprotein J, which further stabilizes LD-associated capsid protein and NS5A, thereby facilitating virion assembly (Lin et al., 2014). Cellular CD2 associated protein (CD2AP) also regulates HCV assembly by interacting with HCV NS5A while modulating LD biogenesis at the same time (Li, 2017). Dissociation of HCV capsid protein from LDs has no effect on viral RNA replication but decreases production of infectious virions, indicating that LDs either directly provide a platform for HCV assembly or facilitate transport of the capsid protein from RNA translation/replication to the assembly sites (Boulant et al., 2007, 2008; Miyanari et al., 2007). Additionally, during chronic HCV infection, LDs in liver tissues increase in number and size, causing pathological accumulation of liver lipids, also known as hepatic steatosis. The interaction between the HCV capsid protein and LDs is critical for this development. An LD membrane protein, perilipin 3, regulates the capsid-induced steatosis, indicating host LD-associated proteins as an effective preventive measure of HCV-induced pathology (Ferguson et al., 2017).

The DENV capsid protein also interacts with LDs but in a mechanistically distinct manner as compared to HCV. DENV capsid protein accumulates on the surface of LDs via its center domain and the N-terminal disordered region (Samsa et al., 2009; Martins et al., 2012). Additionally, the binding between DENV capsid protein and LDs may also be attributed to the association between capsid protein and LD membrane protein perilipin 3 in a potassium ion-dependent fashion. Changing the concentration of potassium ion concentration regulates the binding and release of capsid protein from LDs. This phenomenon indicates that DENV may manipulate specific intracellular ion concentrations to favor viral replication (Carvalho et al., 2012). HCV may use the same potassium ion-dependent strategy to interact with LDs via its p7 and NS5A proteins (Carvalho et al., 2012). Contrary to DGAT1-dependent trafficking to LDs, the DENV capsid protein utilizes host Golgi-specific brefeldin A-resistance guanine nucleotide exchange factor 1 (GBF1)-ARF-COPI pathway to localize to the surface of LDs (Iglesias et al., 2015; Figure 2B). Similar to HCV infection, inhibiting the association between DENV capsid protein and LDs results in attenuated infectious virion production but not viral RNA replication, underscoring the function of LDs as a scaffold for DENV assembly through exposure of the protein cationic surface toward the aqueous environment (Carvalho et al., 2012).

DENV and HCV capsid proteins use distinct mechanisms for LD association. The process by which LDs gain or release viral capsid proteins remain unknown. However, current evidence on the involvement of LDs provides several possible targets for developing antiviral approaches (Table 1) (section Targeting LD Metabolism as Antiviral Strategies).

**Table 1:** Examples antiviral strategies against HCV and flaviviruses interfering with lipid metabolism-related processes.

**Step**	**HCV**				**Flaviviruses**			
	**Lipid biogenesis process**	**Inhibitory target**	**Drug/inhibitor**	**References**	**Lipid biogenesis process**	**Inhibitory target**	**Drug/inhibitor**	**References**
Replication	FA synthesis	FASN	C75	Yang et al., 2008	FA synthesis	FASN	Cerulenin, C75	Heaton et al., 2010; Martín-Acebes et al., 2011; Perera et al., 2012; Poh et al., 2012
		ACC	TOFA	Kapadia and Chisari, 2005		ACC	TOFA, MEDICA 16	Merino-Ramos et al., 2016
	FA β-oxidation	DCI	Etomoxir	Rasmussen et al., 2011	FA β-oxidation	CPT-1	Etomoxir	Heaton and Randall, 2010
	Sphingolipids synthesis	SPT	NA808, myriocin, NA255, ISP-1, HPA-12	Sakamoto et al., 2005; Umehara et al., 2006; Aizaki et al., 2008; Amemiya et al., 2008; Weng et al., 2010; Hirata et al., 2012; Katsume et al., 2013	Sphingolipids synthesis	SMase	D609, MS-209	Martín-Acebes et al., 2016a
						SPT	Myriocin	Aktepe et al., 2015
						CerS	Fumonisin B1	Aktepe et al., 2015
						SphK	SKI/SK1-II	Carr et al., 2013; Clarke et al., 2016
	CHO synthesis	OSBP	OSW-1	Wang et al., 2014	CHO synthesis	SCP-2	SCPI-1	Fu et al., 2015
		PI4KA	AL-9	Wang et al., 2014		SQS	Zaragozic acid A	Rothwell et al., 2009
		SREBPs	BAPTA-AM, pyrrolidine dithiocarbamate	Waris et al., 2007		HMGCS	Hymeglusin	Rothwell et al., 2009
		PI3K	LY294002	Waris et al., 2007; Park et al., 2009		OSC	U18666A	Poh et al., 2012
		CHO	MβCD	Sagan et al., 2006		HMGCR	Lovastatin, paravastatin, hymeglusin, fluvastatin	Mackenzie et al., 2007; Rothwell et al., 2009; Soto-Acosta et al., 2013
		HMGCR	25-hydroxycholesterol, cerulenin, lovastatin, simvastatin, mevastatin, fluvastatin	Ye et al., 2003; Sagan et al., 2006; Amemiya et al., 2008; Delang et al., 2009				
						S1P	PF-429242	Uchida et al., 2016
						DHCR7	AY-9944	Mackenzie et al., 2007
		GGTase I	GGTI-286	Sagan et al., 2006		GGTase I	GGTI-298	Mackenzie et al., 2007
	Glycosphingolipids synthesis	FAPP2	NB-DNJ, PDMP	Khan et al., 2014	Lipophagy	PI3K	3-methyladenine	Heaton and Randall, 2010
	TAG and CHO synthesis	PPARα	BA	Lyn et al., 2009				
	Lipid biosynthesis	AMPK	Metformin, AICAR, A769662	Mankouri et al., 2010				
Assembly	LD-viral capsid protein binding	DGAT1	DGAT1 inhibitor	Herker et al., 2010; Harris et al., 2011	LD-viral capsid protein binding	Perilipin 3	Ouabain	Carvalho et al., 2012
		MAPK, PLA2G4A	U0126	Menzel et al., 2012		MAPK, PLA2G4A	U0126	Menzel et al., 2012
	CHO synthesis	SRB1	PERL	Pollock et al., 2010		GBF1	Brefeldin A	Iglesias et al., 2015
	LDs formation	IKK	Wedelolactone, Inhibitor XII	Li et al., 2013				
		SKI-1/S1P	Spn4A-RRLL	Olmstead et al., 2012				
	LDs redistribution	Microtubule	Nocodazole	Boulant et al., 2008				
Budding	VLDL pathway, LDs lipids transfer	Cyclophilins	NIM811	Anderson et al., 2011	Sphingolipids synthesis	Smase	Amitriptyline	Tani et al., 2010
	Apolipoproteins	MTP	BMS-200150	Perlemuter et al., 2002; Gastaminza et al., 2008		nSmase2	GW4869	Menzel et al., 2012; Martín-Acebes et al., 2014
		MTP/ ACAT2/ HMGCR	Naringenin	Nahmias et al., 2008				

*Underlined, antiviral strategies targeting host lipids and lipid biogenesis processes that directly relate to LDs*.

### Usage of LD as an energy reservoir during viral life cycle

Replication of the viral genome is an energy-consuming process. In HCV infected cells, cytoplasmic ATP levels decrease dramatically, as a result of active energy consumption. Meanwhile, elevated ATP levels at replication compartments within infected cells have also been reported (Ando et al., 2012). This would involve either incorporation of ATP-generating machinery into the membrane-associated replication site, or transport of ATP though membrane-to-membrane communication between mitochondria and replication compartments (Ando et al., 2012). The C terminus of *Flaviviridae* NS3 encodes a DExH/D-box RNA helicase that functions to unwind dsRNA molecules through ATP-hydrolysis (Tai et al., 1996; Dumont et al., 2006). Many of the cellular signaling events activated during viral infection are also regulated by ATP levels (Hardie, 2011). Given the highly reduced and hydrophobic lipids at the core, LDs serve as an efficient storage for energy (Walther and Farese, 2012). FA hydrolysis releases 2.5 times more ATPs per gram compared to glucose, which provides a tremendous reservoir for supplying energy during viral replication. Not surprisingly, many other pathogens also manipulate LD metabolism to acquire fuel for replication.

Energy stored in LDs is released through lipolysis. Mobilization of TAG stores from LDs by lipases produces significant amounts of FFAs that can be used in β-oxidation, generating ATP and other intermediates for the cell. In addition to lipolysis, an alternative route through autophagy, commonly referred to as lipophagy, can also take up and deliver LDs to lytic compartments for lipid hydrolysis (see Mobilization of Lipids from LDs) (Wang, 2016).

A model proposed by Randall and Heaton suggested that DENV infection triggers lipophagy to deplete LDs, releasing FFAs. DENV also induces cellular β-oxidation to consume the FFAs released from lipophagy for energy production. Exogenously supplemented FAs can replace the need for lipophagy during DENV replication, suggesting that flaviviruses manipulate cellular lipid metabolism to create an environment that favors virus replication (Heaton and Randall, 2010). Our own data support this model. AUP1, a monotopic membrane protein localized to both LDs and ER membranes, was identified as a key component in DENV biogenesis. Expression of AUP1 was up-regulated during DENV infection and was found to be necessary for virus-triggered lipophagy to proceed (Zhang et al., 2016). The requirement of lipophagy during other flavivirus infections is still to be investigated.

Virus-induced lipophagy for energy production remains unclear in the context of HCV infection. HCV uses membranes of autophagic vacuoles for viral RNA replication. The induction of autophagosomes is nutrient starvation-independent. An impaired autophagy pathway results in attenuated virion production (Dreux et al., 2009; Sir et al., 2012). Proteomic and lipidomic studies showed an up-regulation of lipogenic enzymes and proteins related to β-oxidation, such as 3,2-trans-enoyl-CoA isomerase (DCI) (Diamond et al., 2010). In line with this study, DCI was reported to be essential for productive HCV infection through regulation of mitochondrial FA oxidation (Rasmussen et al., 2011). Another microarray analysis revealed a down-regulation of genes involved in degradation and oxidation of FAs, and an elevation of genes that control metabolism and transport of FAs (Blackham et al., 2010). Although a direct experimental evidence of lipophagy induced by HCV is still missing, data from several indirect sources strongly suggest the utilization of cellular pathways for β-oxidation of FFAs.

### Manipulation of LD reserves during viral life cycle

Apart from providing FFAs for β-oxidation during *Flaviviridae* infection, LDs also function as a reservoir for lipids that are essential for viral replication.

*Flaviviridae* replication organelles consist of FAs, specific phospholipids, sphingolipids, and cholesterol (Heaton et al., 2010; Perera et al., 2012; Paul et al., 2013; Martín-Acebes et al., 2016a). While DENV obtains FAs by breakdown of LDs via lipophagy (Heaton and Randall, 2010), HCV controls the transcriptional induction of lipid biosynthetic and related genes through SREBP signaling (Olmstead et al., 2012). HCV infection activates the SREBP precursor that localizes to the ER, and triggers its trafficking to the Golgi. Thereafter, the SREBP precursor is proteolytically processed by site 1 protease (S1P) and S2P at Golgi, releasing its N-terminal fragment that is transported into the nucleus and initiates transcription of lipogenic factors such as FASN and 3-hydroxy-3-methylglutaryl CoA (HMGCoA). The 3′ untranslated region of the HCV RNA genome with DEAD box polypeptide 3 X-linked (DDX3X) further activates IκB kinase (IKK)-α, which translocates to the nucleus and stimulates SREBP transcriptional activity, thus modulating LD biogenesis (Olmstead et al., 2012; Li et al., 2013).

HCV replication organelles use cholesterol as a structural component (Romero-Brey et al., 2012; Paul et al., 2013). Cellular oxysterol-binding protein (OSBP) and phosphatidylinositol 4-kinases (PI4KA) facilitate trafficking of cholesterol to the HCV-rearranged membrane-like structures during replication, highlighting the need for both factors in supporting HCV replication (Wang et al., 2014). OSBPs are speculated to be sterol carriers and might function to transport sterols out of the ER and incorporate them into LDs in a phosphatidylinositol 4-phosphate (PI(4)P)-dependent manner. Sterols and cholesterol are exchanged by OSBP at the ER-Golgi interface (Mesmin et al., 2013). OSBP-related protein 2 that resides on the surface of LDs may also participate in the process of lipid exchange (Hynynen et al., 2009). Notwithstanding its cellular function, the activity of OSBP appears to be dispensable for DENV replication (Hynynen et al., 2009). DENV replication is regulated by endogenous cholesterol production that is controlled by mevalonate (diphospho) decarboxylase (MVD) and exogenous cholesterol uptake (Rothwell et al., 2009). Similarly, WNV also hijacks cellular cholesterol and redistributes it to viral RNA replication compartments (Mackenzie et al., 2007).

Besides cholesterol, sphingomyelin is another essential membrane component of HCV replication organelles. An active role for sphingolipids in HCV RNA replication has been reported. Sphingomyelin enhances binding of the RNA dependent RNA polymerase NS5B to the template RNA and is therefore important for HCV replication (Weng et al., 2010; Hirata et al., 2012). Expression of genes that encode sphingomyelin synthases 1 and 2 is up-regulated upon HCV infection, resulting in enhanced synthesis of sphingomyelin (Hirata et al., 2012). Dynamic pools of sphingomyelin were observed in LDs, with the high affinity sphingomyelin-binding protein ADRP on the surface of LDs (McIntosh et al., 2010). It is likely that LDs participate in the biogenesis of sphingolipids necessary for HCV replication.

In addition to consumption of lipids that are stored in LDs, HCV can also obstruct the turnover of LDs to establish a microenvironment that is more favorable to viral infection. Release of infectious HCV particles relies on secretion of hepatic very low-density lipoprotein (VLDL)—a TAG-rich lipoprotein. For hijacking VLDL secretion, HCV inhibits the function of the putative TAG lipase, arylacetamide deacetylase (AADAC), thus, further impairing TAG lipolysis (Nourbakhsh et al., 2013). Moreover, HCV capsid protein that localizes to LDs through the activity of DGAT1 (Harris et al., 2011), restrains lipolysis of TAG by interacting with ATGL and its activator comparative gene identification-58 (CGI-58) (Camus et al., 2014).

As with LD association, distinct strategies are employed by HCV and DENV for mobilizing lipids within LDs, hence providing insights into LD catabolism and cellular factors as possible targets (Table 1).

## Targeting LD metabolism as antiviral strategies

Although viruses of the *Flaviviridae* family cause severe human diseases, there are currently no clinically approved drugs available for treatment against them, other than for HCV. Historically, the development of antiviral therapy has largely focused on directly targeting viral components involved in multiple stages of the virus life cycle.

Entry of flaviviruses is mediated by fusion of the viral envelope (E) protein with the host membrane. Blocking virus entry via targeting the viral E protein offers a means to suppress the onset of infection. A few heterocyclic compounds, such as compound 6, NITD-448 and P02, have been identified to directly bind to the hydrophobic pocket of viral E protein and block its conformational change, which is essential for virus-host fusion (Modis et al., 2003, 2004; Zhou et al., 2008; Poh et al., 2009; Wang et al., 2009). Due to the multifunctional nature of the E protein, its inhibitors may potentially block multiple steps in the viral life cycle, including entry and virion assembly/maturation. More importantly, these inhibitors can exert their effect through direct binding to virions without the need to cross the hydrophobic membrane bilayer and be delivered into infected cells. However, due to the complexity and high variability of flaviviral E protein, it is challenging to develop pan-serotype inhibitors (Wang and Shi, 2015).

During replication, the viral genome is translated into a single polyprotein which is cleaved into individual proteins by a viral protease complex. Since polyprotein processing is a prerequisite for viral replication and assembly, these virally encoded proteases are one of the most attractive antiviral targets (Chambers et al., 1990, 1993; Luo et al., 2015). Two HCV NS3/4A serine protease inhibitors, boceprevir and telaprevir, have been approved in combination with PEG-interferon plus ribavirin for treatment of chronic HCV genotype 1 (Ghany et al., 2011). Recent study by Shiryaev and colleagues have identified a group of small molecule antiviral inhibitors that interfering with the productive fold of the NS2B cofactor in the two-component protease, inhibit its cleavage activity and therefore suppress ZIKV infection. The most potent inhibitor NSC157058 was shown to inhibit ZIKV infection in both cultured hfNPCs and mice without significant toxicity (Shiryaev et al., 2017). Despite these advances, resistance to protease inhibitors can occur rapidly, especially for chronic infections such as HCV due to the genetic variability of the virus and high mutation rate (Rong et al., 2010; Wu et al., 2013). Another concern in developing protease-based antiviral therapy is toxicity. Similarities in viral and host cellular serine proteases would presumably create problems in specificity while targeting the virus.

The flaviviral NS3 RNA helicase is located adjacent to the C terminal of the NS3 protease (Luo et al., 2008). The RNA helicase is believed to be required for several different functions such as initiation of RNA synthesis, separating dsRNA structures formed during viral RNA synthesis and as a translocase that eliminates proteins bound to the viral RNA (Sampath and Padmanabhan, 2009). Viruses with a mutated NS3 helicase are unable to replicate properly (Matusan et al., 2001). Several RNA helicase inhibitors have been identified. The antiparasitic drug ivermectin was shown to inhibit WNV, YFV, and DENV at submicromolar levels, and a small molecule inhibitor ST-610 was found to potently and selectively inhibit all four serotypes of DENV *in vivo* (Mastrangelo et al., 2012; Lim et al., 2013). However, due to a lack of specific binding pockets for RNA and NTPs, molecules that target the RNA helicase via these binding sites might also non-selectively bind to other cellular proteins with helicase/NTPase activities, resulting in significant toxicity (Luo et al., 2015).

The NS5 RNA-dependent RNA polymerase (RdRp) is the most conserved amongst the flavivirus proteins, and is essential for viral RNA synthesis. Since host cells lack these enzymes, the specificity makes them one of the most promising and intensively studied classes of antiviral targets. RdRp can be targeted by non-nucleoside inhibitors (NNIs) and nucleoside/nucleotide analog inhibitors (NIs) (Malet et al., 2008). NNIs directly target the binding pocket of the polymerase and block its conformational change from its inactive to active form (Biswal et al., 2005). Although a number of NNI candidates for HCV are under clinical development, there hasn't been any FDA approved NNIs for flaviviruses yet. The major challenge in the use of NNIs in antiviral therapy is the structural variability of the binding pockets across different serotypes or genotypes as well as the resistant mutation in or near the binding pocket which results in resistance to the NNIs (Sofia et al., 2012). NIs have been widely used in clinics for treatment of hepatitis, HIV and herpesvirus infections (Jordheim et al., 2013; Menéndez-Arias et al., 2014). Compared to other classes of inhibitors, NIs have a higher threshold for developing resistance, and a relatively broad-antiviral spectrum due to the relatively conserved polymerase structure (Delang et al., 2011; Lim et al., 2013). Unlike NNIs which directly bind to RNA polymerase, NIs have to convert into its triphosphate form inside cells by host kinases before exerting their antiviral effects (Stein and Moore, 2001). However, the kinase activity varies significantly in different cell types/hosts, causing variable efficacy of the same NI. Another major issue associated with NIs is the unpredictable toxicity *in vitro*. Although the toxicity of NIs is often associated with the inhibition of mitochondrial polymerases (Arnold et al., 2012), other mitochondrial perturbations may also attribute to toxicity (Selvaraj et al., 2014).

The N-terminal domain of NS5 contains one methyltransferase (MTase) that catalyzes guanine N-7 and ribose 2′-*O*-methylations using S-adenosylmethionine (SAM) as a methyl donor during viral cap formation (Zhou et al., 2007). Non-selective competitive inhibitors, such as S-adenosylhomocysteine and sinefungin bind to SAM binding sites and inhibit its function (Boldescu et al., 2017). Using virtual screening, a group of small compound molecules have been identified with broad-spectrum activity against the MTase proteins of multiple flaviviruses, including DENV2, DENV3, and YFV (Brecher et al., 2015). Apart from the most important antiviral targets such as E protein, NS3 protease and NS5 polymerase, other viral targets such as capsid protein, NS1 and NS4 proteins are also under evaluation. The details of different viral targets have been reviewed elsewhere (Boldescu et al., 2017; García et al., 2017).

Due to extensive dependence of viruses (replication, assembly, and budding) on host LDs, the interface of virus-host interactions with LDs and/or LD metabolism provides a rich source for potential antiviral interventions (Table 1). First, targeting host factors may produce potential broad-spectrum activity against multiple viral infections due to similar intracellular pathways employed by viruses within the same genus or family. Second, given the high replication and mutation rates of viruses, long-term antiviral therapy against chronic infections inevitably selects for the resistant variants which alter the drug target and therefore are less susceptible to the inhibitory effects of the treatment. The resistant mutants eventually become the dominant species and lead to treatment failure and persistent infection. Development of drug-resistance has become a major challenge with direct-acting antivirals when treating chronic infections (Rong et al., 2010). Unlike viral elements, host cellular factors are much less prone to mutation; thus targeting host lipid metabolism provides an attractive approach for long-term treatment of diseases caused by viral infection. However, since LDs play a role in lipid metabolism *in-vivo*, manipulating a major metabolic pathway may have a more pleiotropic impact on cellular homeostasis (Georgel et al., 2010). Such consequences need to be carefully assessed to hit the right balance between causing host toxicity while preventing viral pathogenesis. Third, several inhibitors targeting host lipid metabolic pathways are well characterized, which can greatly accelerate the process of drug development. Moreover, targeting specific steps of LD biosynthesis, distribution, trafficking, and metabolism which viruses routinely exploit, allows us to design antiviral strategies with an enhanced therapeutic window. For example, triglyceride-synthesizing enzyme DGAT1 has been identified as an important host factor which is required for trafficking of viral capsid protein to LDs, facilitating early steps of viral assembly. Of note, RNAi-mediated silencing of DGAT1 resulted in impaired viral particle production without affecting LD composition (Herker et al., 2010). Currently, novel classes of pharmacological inhibitors targeting DGAT1 have been developed for clinical applications (DeVita and Pinto, 2013). In addition, regulating enzymes in the FA synthesis pathway has been shown to inhibit production of different viruses. C75, a FA synthase inhibitor, displayed a strong inhibitory effect on HCV replication (Yang et al., 2008), DENV production (Samsa et al., 2009), as well as WNV and YFV replication (Martín-Acebes et al., 2011) without causing significant toxicity to host cells. A series of chemical probes (ML-206, ML-219 and ML-220) has been shown to reduce the biogenesis and consumption of LDs without toxicity to mammalian cells (Boxer et al., 2013). These probes may prove to be beneficial in inhibiting virus production. A noteworthy and indirect strategy to interrupt the association between virus and LDs during viral replication and assembly is to target involved viral proteins. During the biosynthesis of the HCV polyproteins, an internal signal sequence between the capsid protein and envelope protein E1 can be preceded by cellular signal peptide peptidase (SPP). This process releases the capsid protein from the ER, followed by its transport to LDs. SPP inhibitor (Z-LL)_2_-ketone abolishes the cleavage of capsid protein by SPP and thereby inhibits production of infectious HCV (McLauchlan et al., 2002).

Ideally, antiviral treatments should exert their effects as early as possible after infection. This is particularly true for acute flaviviral infections such as DENV. Targeting intracellular host factors, however, is perhaps less effective in preventing the onset of an infection compared to other inhibitors, which block viral entry. The advantages and disadvantages of antiviral strategies against HCV and flaviviruses by targeting viral components and host factors including those involved in LD metabolism are summarized in Table 2.

**Table 2:** Comparison of advantages and disadvantages of different antiviral strategies against HCV and flaviviruses.

	**Host Target**		**Viral Target**			
	**Host factors in lipid metabolism**	**Other cellular factors (host protease, glucosidase, kinases)**	**Viral entry**	**Viral proteasome**	**RNA helicase**	**RdRp**
Advantages	Higher barrier to resistance Broad-spectrum antiviral effects, e.g., Statin Effective control of emerging and novel pathogens Fast development process with known inhibitors Can impair both genome replication and particle morphogenesis	Higher barrier to resistance Broad-spectrum antiviral effects, e.g., DNJ Effective control of emerging and novel pathogens Fast development process with known inhibitors Target different steps of viral life cycle Target specific manifestations, e.g., DHF/DSS	Effective in early steps of viral life cycle Fast inhibition for acute infection No need for penetration into the host cells Blocking multiple steps of viral cycle (entry and virion assembly/maturation)	Broad-spectrum of antiviral activity Potential of combination therapy	Broad-spectrum of antiviral activity	**Non-nucleoside inhibitors (NNIs)** Lower off-target effects Lower cytotoxicity **Nucleoside/nucleotide analog inhibitors (NIs)** Higher barrier to resistance Broad spectrum of antiviral activity Lower off-target effects
Disadvantages	Not effective in early steps of viral life cycle (entry and fusion) Enhanced cellular toxicities, e.g., PI4KIIIα inhibitors	Enhanced cellular toxicities, e.g., NITD008 and Balapiravir Drug resistance induced by viral substrate mutation, e.g., kinase inhibitor AZD0530	Low barrier to resistance Difficult to develop pan-serotype inhibitors	Low barrier to resistance Cross inhibition against human enzymes	Enhanced toxicity due to lack of specific binding pockets for RNA and NTP binding sites	**Non-nucleoside inhibitors (NNIs)** Low barrier to resistance Difficult to develop pan-serotype inhibitors **Nucleoside/nucleotide analog inhibitors (NIs)** Unpredictable structure-activity relationship Varied efficacy across different cell types and hosts Unpredictable toxicity *in vivo*

## Conclusion

Despite being an immense global health problem, there are no affordable and efficient prophylactic or therapeutic treatments for some pathogenic flaviviruses. It is imperative to have alternative therapeutic strategies of inhibiting specific steps in the intracellular virus life cycle to combat infection. Viruses from the *Flaviviridae* family often cause perturbations in cellular energy and lipid homeostasis during infection. This has been reported for DENV, WNV, and HCV infection. Therefore, targeting cellular LDs offers possibilities for such interventions, including inhibition of lipid metabolism and disruption of interactions with viral components. Although knowledge on the participation of LDs during infection of HCV and flaviviruses has significantly progressed, comparative studies that aim to determine the shared or specific requirements of LD components for these pathogens are still lacking. In addition, much of the information available is from *in-vitro* studies, while the *in-vivo* relevance remains unexplored. Therefore, a more comprehensive understanding of the molecular biology of viruses and their dependence on host LD metabolism is of utmost priority for development of broad-spectrum and specific anti-flaviviral strategies.

## Author contributions

JZ and YL drafted the manuscript and contributed equally to this work. SS supervised, evaluated, and edited the manuscript.

### Conflict of interest statement

The authors declare that the research was conducted in the absence of any commercial or financial relationships that could be construed as a potential conflict of interest.

## Abbreviations

769662: 6,7-Dihydro-4-hydroxy-3-(2′-hydroxy[1,1′-biphenyl]-4-yl)-6-oxo-thieno[2,3-b]pyridine-5-carbonitrile
AADAC: arylacetamide deacetylase
ACAT: acyl-CoA, cholesterol acyltranserases
ACC: acetyl coenzyme a carboxylase
ADRP: adipose differentiation-related protein
AICAR: aminoimidazole carboxamide ribonucleotide
AMPK: 5′ AMP-activated protein kinase
ApoB100: apolipoprotein B100
ARF: ADP-ribosylation factor
ARF1-COP I: ADP-ribosylation factor-coat protein I
ATGL: adipose triglyceride lipase
AUP1: ancient ubiquitous protein 1
AY9944: trans-1,4-bis(2-Chlorobenzylaminoethyl) cyclohexane dihydrochloride
BA: 2-chloro-5-nitro-N-(pyridyl)benzamide
BAPTA-AM: 1,2-Bis(2-aminophenoxy)ethane-*N,N,N*′*N*′-tetraacetic acid tetrakis(acetoxymethyl ester)
BMS-200150: 2-[1-(3,3-diphenylpropyl)-4-piperidinyl]-2,3-dihydro-1H-isoindol-1-one
C75: 4-Methylene-2-octyl-5-oxotetrahydrofuran-3-carboxylic acid
CD2AP: CD2 associated protein
CE: cholesterol ester
CerS: ceramide synthase
CGI58: comparative gene identification-58
CHO: cholesterol
CMA: chaperone-mediated autophagy
COP: coat protein
CPT-1: carnitine palmitoyltransferase 1
D609: tricyclodecan-9-yl-xanthogenate
DAG: diacylyglycerol
DCI: 3,2-trans-enoyl-CoA iosmerase
DDX3X: DEAD box polypeptide 3 X-linked
DENV: dengue virus
DGAT: diacylglycerol acyltransferases
DHCR: dehydrocholesterol reductase
DHF: dengue hemorrhagic fever
DMV: double-membrane vesicle
DNJ: 1-deoxynojirimycin
dsRNA: double-stranded RNA
DSS: dengue shock syndrome
E protein: envelope protein
ER: endoplasmic reticulum
ERAD: ER-associated degradation
ERGIC: ER–Golgi intermediate compartment
FAPP2: Golgi-associated four-phosphate adaptor protein 2
FASN: fatty acid synthase
GBF1: golgi-specific brefeldin A-resistance guanine nucleotide exchange factor 1
GGTase I: geranylgeranyltransferase
GGTI-286: *N*-4-[2(*R*)-Amino-3-mercaptopropyl]amino-2-phenylbenzoyl-(L)-leucine methyl ester
GGTI-298: *N*-4-[2(*R*)-Amino-3-mercaptopropyl]amino-2-naphthylbenzoyl-(L)-leucine methyl ester trifluoroacetate salt
GW4869: N,N′-Bis[4-(4,5-dihydro-1H-imidazol-2-yl)phenyl]-3,3′-*p*-phenylene-bis-acrylamide dihydrochloride
HCV: hepatitis C virus
HMGCoA: 3-hydroxy-3-methylglutaryl CoA
HMGCR: 3-hydroxy-methyglutaryl-Coenzyme A reductase
HMGCS: hydroxymethylglutaryl-CoA synthase
HSL: hormone-sensitive lipase
IKK: IkB kinase
JEV: Japanese encephalitis virus
LD: lipid droplet
LY294002: 2-(4-Morpholinyl)-8-phenyl-4*H*-1-benzopyran-4-one
MAPK: mitogen-activated protein kinases
MEDICA: 16:3,3,14,14-Tetramethylhexadecanedioic acid
MGL: monoglyceride lipase
MS-209: dofequidar fumarate
MTase: methyltransferase
MTOC: microtubule-organizing center
MTP: microsomal triglyceride transfer protein large subunit
MVD: mevalonate (diphospho) decarboxylase
MβCD: methyl-β-cyclodextrin
NIM811: *N*-methyl-4-isoleucine cyclosporine
NB-DNJ: N-Butyldeoxynojirimycin
NIs: nucleoside/nucleotide analog inhibitors
NNIs: non-nucleoside inhibitors
NS: non-structural
nSMase2: neutral sphingomyelinase 2
OSBP: oxysterol-binding protein
OSC: oxidosqualene cyclase
OSW-1: 3β,16β,17α-trihydroxycholest-5-en-22-one 16-*O*-(2-*O*-4-methoxybenzoyl-β-*D*-xylopyranosyl)-(1 → 3)-(2-*O*-acetyl-α-l-arabinopyranoside)
PC: phosphatidylcholine
PDMP: D,L-*threo*-1-phenyl-2-decanoylamino-3-morpholino-1-propanol
PE: phosphatidylethanolamine
PERL: polyunsaturated ER liposomes
PF-429242: 4-[(Diethylamino)methyl]-*N*-[2-(2-methoxyphenyl)ethyl]-*N*-(*3R*)-3-pyrrolidinyl-benzamide
PI3K: phosphatidylinositide 3-kinases
PI4KA: phosphatidylinositol 4-kinases
PI4P: phosphatidylinositol 4-phosphate
PLA2G4A: cytosolic phospholipase A2
PNPLA5: patatin-like phospholipase domain-containing protein 5
PPARα: peroxisome proliferator-activated receptor α
RdRp: RNA-dependent RNA polymerase
S1P: site 1 protease
SAM: S-adenosylmethionine
SCAP: Sterol regulatory element-binding protein cleavage-activating protein
SCPI-1: [*N*-(4-{[4-(3,4-dichlorophenyl)-1,3-thiazol-2-yl]amino}-phenyl)acetamidehydrobromide]
SCP-2: sterol carrier protein 2
SKI-1: subtilisin/kexin-isozyme-1
Smase: sphingomyelinase
SphK: sphingosine kinase
SPP: signal peptide peptidase
SPT: serine palmitoyltransferase
SQS: squalene synthetase
SRB1: scavenger receptor class B member 1
SREBP: sterol regulatory element–binding protein
TAG: triacylglycerol
TBEV: tick-borne encephalitis virus
TOFA: 5-tetradecyl-oxy-2-furoic acid
U0126: 1,4-Diamino-2,3-dicyano-1,4-*bis*(2-aminophenylthio)butadiene
U18666A: 3β-(2-Diethylaminoethoxy)androst-5-en-17-one
VAP: vesicle-associated membrane protein-associated protein
VLDL: very low-density lipoprotein
WNV: West Nile virus
YFV: yellow fever virus
ZIKV: Zika virus.
